# Dimensionality of the Pittsburgh Sleep Quality Index in the young collegiate adults

**DOI:** 10.1186/s40064-016-3234-x

**Published:** 2016-09-13

**Authors:** Md. Dilshad Manzar, Wassilatul Zannat, M. Ejaz Hussain, Seithikurippu R. Pandi-Perumal, Ahmed S. Bahammam, Doaa Barakat, Nwakile Izuchukwu Ojike, Awad Olaish, D. Warren Spence

**Affiliations:** 1 Department of Biomedical Sciences, College of Health Sciences, Mizan Tepi University (Mizan Campus), Mizan Aman Town, Ethiopia; 2 Centre for Physiotherapy and Rehabilitation Sciences, Jamia Millia Islamia, New Delhi, India; 3 Somnogen Canada Inc, College Street, Toronto, ON Canada; 4 The University Sleep Disorders Center, Department of Medicine, College of Medicine, and National Plan for Science and Technology, College of Medicine, King Saud University, Riyadh, Saudi Arabia; 5 Department of Psychiatry, Faculty of Medicine, Ain Shams University, Cairo, Egypt; 6 Center for Healthful Behavior Change, Department of Population Health, New York University Medical Center, New York, NY USA; 7 323 Brock Ave., Toronto, ON M6K 2M6 Canada

**Keywords:** Confirmatory factor analysis, Exploratory factor analysis, Collegiate, young adults, Model fit, Students

## Abstract

**Purpose:**

To explore and validate the factor structure of the Pittsburgh Sleep Quality Index (PSQI) in young collegiate adults.

**Methods:**

Six hundred university students were initially contacted and invited to participate in a survey of their sleep experience and history. Of this preliminary sample 418 of the students (age = 20.92 ± 1.81 years, BMI = 23.30 ± 2.57 kg/m^2^) fulfilled the screening criteria and ultimately completed the Pittsburgh Sleep Quality Index (PSQI), a self-report survey of respondents’ sleep habits and sleep quality. The students were enrolled in various undergraduate and postgraduate programs at Jamia Millia Islamia, New Delhi, India. Exploratory factor analysis (EFA) investigated the latent factor structure of the scale. Confirmatory factor analysis evaluated both of the models found by EFA.

**Results:**

The Kaiser’s criteria, the Scree test, and the cumulative variance rule revealed that a 2-factor model accounted for most of the variability in the data. However, a follow up Parallel Analysis found a 1-factor model. The high correlation coefficient (r = 0.91) between the two factors of the 2-factor model and almost similar values of the fit indices supports the inference that the PSQI is a unidimensional scale.

**Conclusions:**

The findings validate the 1-factor model of the PSQI in young collegiate adults.

## Background

Difficulties with sleeping are an endemic problem among college students in competitive academic environments (Manzar et al. 2015). Sleep problems are often part of a feedback cycle, being an important result of as well as the cause of many of the challenges of university life. Disrupted sleep has direct effects on the mental alertness, attention span, and cognition of young adults, and consequently can affect their overall health and academic performance. Other sequellae of disturbed sleep are well documented and include, but are not limited to, daytime fatigue, anxiety, stress, depression, sympathetic activity changes, and cardio-vascular problems. These direct health effects have secondary behavioral consequences such as inappropriate impulsivity, impaired social relationships, increased risk-taking behavior, and a greater likelihood of having a motor vehicle accident (Sweileh et al. 2011). The ability to identify the presence of disturbed sleep through valid and easy-to-administer questionnaires thus represents a valuable “early warning system” for counselors and other health professionals who work with students. Such testing instruments can be useful diagnostic tools in the process of identifying those who may be at risk for more serious adjustment problems later, as well as for establishing a program of preventive and therapeutic measures.

The Pittsburgh Sleep Quality index (PSQI) is one of the most widely used sleep diagnostic questionnaire tools. The nineteen self-reported items of the scale are pooled to generate seven component scores, all of which sum to a global score. This global score is a measure of subjective sleep quality for the period of the one month immediately preceding the survey. Many aspects of the validity of the PSQI validity are well established in different age groups, clinical and non-clinical populations, and among those of differing ethnicities and regions of the world (Buysse et al. 1989; Mollayeva et al. 2016; Manzar et al. 2015). However, various studies have shown inconsistencies with respect to the dimensionality of the PSQI as this has been investigated among both general and collegiate samples (Mollayeva et al. 2016; Gelaye et al. 2014; Aloba et al. 2007). These inconsistencies have thus made it difficult to evaluate the applicability of the PSQI generally or among various sub populations such as university students. The present study therefore sought to clarify this issue and to validate the dimensionality of the PSQI in a sample of young collegiate adults.

## Methods

### Study design and subjects

A sample of students at Jamia Millia Islamia, New Delhi, India were recruited and invited to participate in a *semi*-structured sleep survey. Four hundred eighteen participants out of an initial 600 students who were screened and who had been found qualified were given the survey and fully completed it. The subjects were young adults (age = 20.92 ± 1.81 years, BMI = 23.30 ± 2.57 kg/m^2^) with male (n = 198) to female (n = 220) ratio of 0.9. Potential participants who reported any health conditions related to cardiovascular, neurological, or psychiatric disorders, or who had any experience of chronic pain, or any recent history of major injury/surgery, or emotional problems were excluded from the study. The students were enrolled in various undergraduate and postgraduate courses at the university. The average global score of the PSQI was more than 5, i.e. indicative of the presence of clinically significant sleep difficulties. The sample (n = 418) was randomly divided into two equal sub-samples for factor analysis employing cross validation (Cole et al. 2006). Exploratory factor analysis (EFA) was performed on the first sub-sample. The resulting model was tested by confirmatory factor analysis (CFA) on a second sub-sample. The study was approved by the human institutional ethics committee. This is a secondary analysis of the data presented in our previous paper. More details about participant characteristics and methods of data collection are documented therein (Manzar et al. 2015).

### Statistical analysis

The statistical package, SPSS 16.0 (SPSS Inc., Chicago, Illinois) was used. The nineteen items of the PSQI transform non-linearly into seven component scores. Therefore, the factor analysis was performed on the PSQI component scores.

The sample and the PSQI components satisfied conditions of Kaiser–Meyer–Olkin (KMO) (0.754), Bartlett’s test of sphericity (p < 0.001), communality retention criteria (0.40–0.70), anti-image matrix (all values >0.5), and determinant (>0.00001) (Beavers et al. 2013; Williams et al. 2010). Principal component analysis gave an initial estimate of the number of factors. The Kaiser criterion (Eigenvalue >1), cumulative variance rule (>40 %), Scree plot and Parallel Analysis (Monte Carlo PA) with Principal Components and Random Normal Data Generation were employed. Maximum likelihood estimation with direct oblimin rotation was used in the final EFA. The least value of the loading retained was 0.39 with no cross-over loadings above 0.4 (Williams et al. 2010).

The PSQI components are ordered categorical variables and moreover their distribution had issues of skewness and kurtosis (Table 1). Therefore, Maximum likelihood extraction with bootstrapping to smooth non-normality with standardized estimates of factor loading was employed for CFA (Bollen and Stine 1992; Nevitt and Hancock 2000). Multiple fit indices from different classes were used for the test of adequate fitness and the selection of a better fit model (Marsh et al. 1996). A non-significant χ^2^ and χ^2^/df ratio of less than 2 suggested an acceptable fit between the model and the data (Ullman 2001). The root mean square residual (RMR) value of up to 0.05 indicated good fit. A comparative fit index (CFI) of at least 0.95, and a root mean square error of approximation (RMSEA) of less than 0.05 indicated good fit. The Akaike information criterion (AIC) was employed as a relative measure of fit between models. Its lesser value indicated a better model fit. The goodness of fit index (GFI) and adjusted goodness of fit index (AGFI) (>0.9) both indicated a good fit (Hu and Bentler 1999).

**Table 1:** Descriptive statistics of the Pittsburgh Sleep Quality Index: Confirmatory factory analysis sub-sample in the collegiate young adults

Pittsburgh Sleep Quality Index (PSQI) components	Mean ± SD	Skewness ± SE	Kurtosis ± SE
PSQI component of sleep duration	1.04 ± 0.935	0.566 ± 0.168	−0.568 ± 0.335
PSQI component of sleep disturbances	1.14 ± 0.527	0.550 ± 0.168	1.412 ± 0.335
PSQI component of sleep latency	1.18 ± 0.947	0.325 ± 0.168	−0.842 ± 0.335
PSQI component of daytime dysfunction	0.88 ± 0.820	0.700 ± 0.168	−0.018 ± 0.335
PSQI component of sleep efficiency	0.17 ± 0.496	3.437 ± 0.168	12.871 ± 0.335
PSQI component of overall sleep quality	0.99 ± 0.676	0.678 ± 0.168	1.262 ± 0.335
PSQI component of sleep medication	0.08 ± 0.385	5.871 ± 0.168	37.154 ± 0.335
Multivariate			59.182 ± 1.527

*SD* standard deviation, *SE* standard error

## Results

Both the sub-samples had a similar range (0–15 and 0–16 respectively) and mean (5.65 ± 2.94 and 5.46 ± 2.77 respectively) of the PSQI global score. Inter-PSQI component correlations were similar in the two sub-samples. The sub-samples had a 0–3 range of distribution for each of the PSQI component scores.

### Exploratory factor analysis

**Fig. 1:**
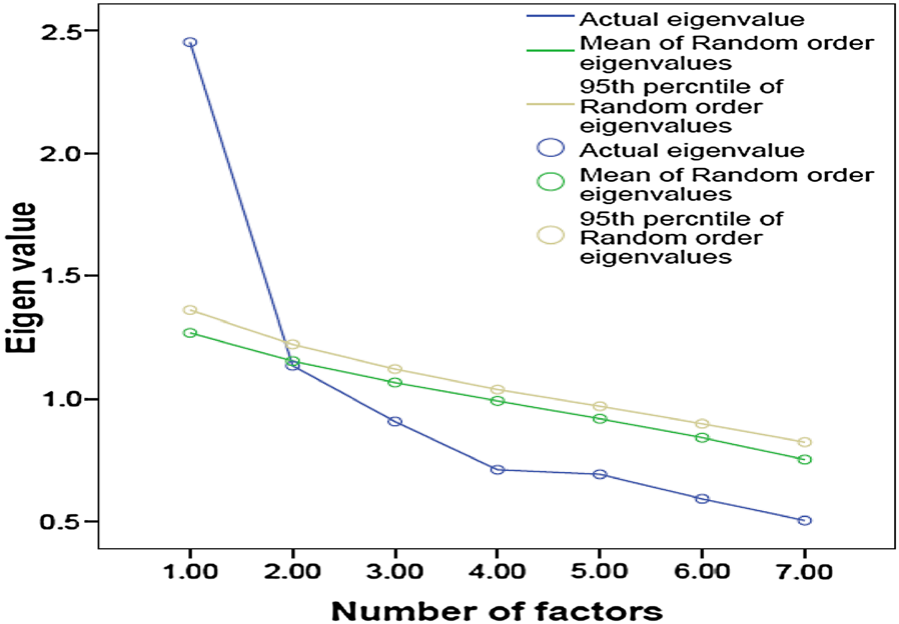
Parallel Analysis Sequence plot of the Pittsburgh Sleep Quality Index in the collegiate young adults

Kaiser’s criteria, the Scree test: the point of inflexion of the actual Eigenvalue plot (blue curve; Fig. 1) and cumulative variance rule revealed the existence of a 2-factor model (Beavers et al. 2013; Williams et al. 2010). Both the factors were named according to the relative loading contributions from the PSQI components for sleep latency. These were named sleep quality, and sleep efficiency because these had maximum loading from the PSQI components of sleep quality and habitual sleep efficiency respectively. The loadings of the PSQI complement components retained for performing CFA ranged from 0.77 (the PSQI component of sleep quality) to 0.39 (the PSQI component of sleep latency). The PSQI component of sleep latency had poor loadings on either of the factors. However, it was adjudged to load on the sleep efficiency factor because of its relatively higher load on this factor (Table 2). The correlation between the latent factors was strong (0.63) (Cohen 1988), and accounted for a cumulative variance of 51.27 % (Beavers et al. 2013; Williams et al. 2010). The Parallel Analysis revealed 1-factor for the PSQI (Table 3; Fig. 1); the actual Eigenvalue for the second factor was less than the 95th percentile of the random ordered Eigenvalue.

**Table 2:** Factor matrix of the 2-Factor model of the Pittsburgh Sleep Quality Index in the collegiate young adults

Pittsburgh Sleep Quality Index (PSQI) component	Sleep quality^a^	Sleep efficiency^a^	Communality (h2)
PSQI component of overall sleep quality	.723	.104	.416
PSQI component of daytime dysfunction	.468	−.019	.513
PSQI component of sleep duration	.404	−.020	.502
PSQI component of sleep medication	−.160	.659	.508
PSQI component of sleep disturbances	.191	.502	.387
PSQI component of sleep efficiency	.121	.397	.620
PSQI component of sleep latency	.344	.387	.644
Percentage of total variance (%)	35.045	16.228	.416

Exploratory Factor analysis (EFA) with maximum likelihood extraction and direct oblimin rotation method was performed

^a^Latent factors derived from EFA

**Table 3:** Parallel Analysis (Monte Carlo PA) Output of the Pittsburgh Sleep Quality Index in the collegiate young adults

Number of factors	Actual eigenvalue from PCA	Random order eigenvalues (means)	Random order eigenvalues (95th percentile)
1	2.45	1.27	1.36
2	*1.14*	1.15	*1.22*
3	.91	1.07	1.12
4	.71	.99	1.04
5	.69	.92	.97
6	.59	.84	.90
7	.50	.75	.82

Italic values indicate the actual Eigenvalue (1.14) for the second factor was less than the 95th percentile of the random ordered Eigenvalue (1.22)

*PCA* principal component analysis

### Confirmatory factor analysis

**Fig. 2:**
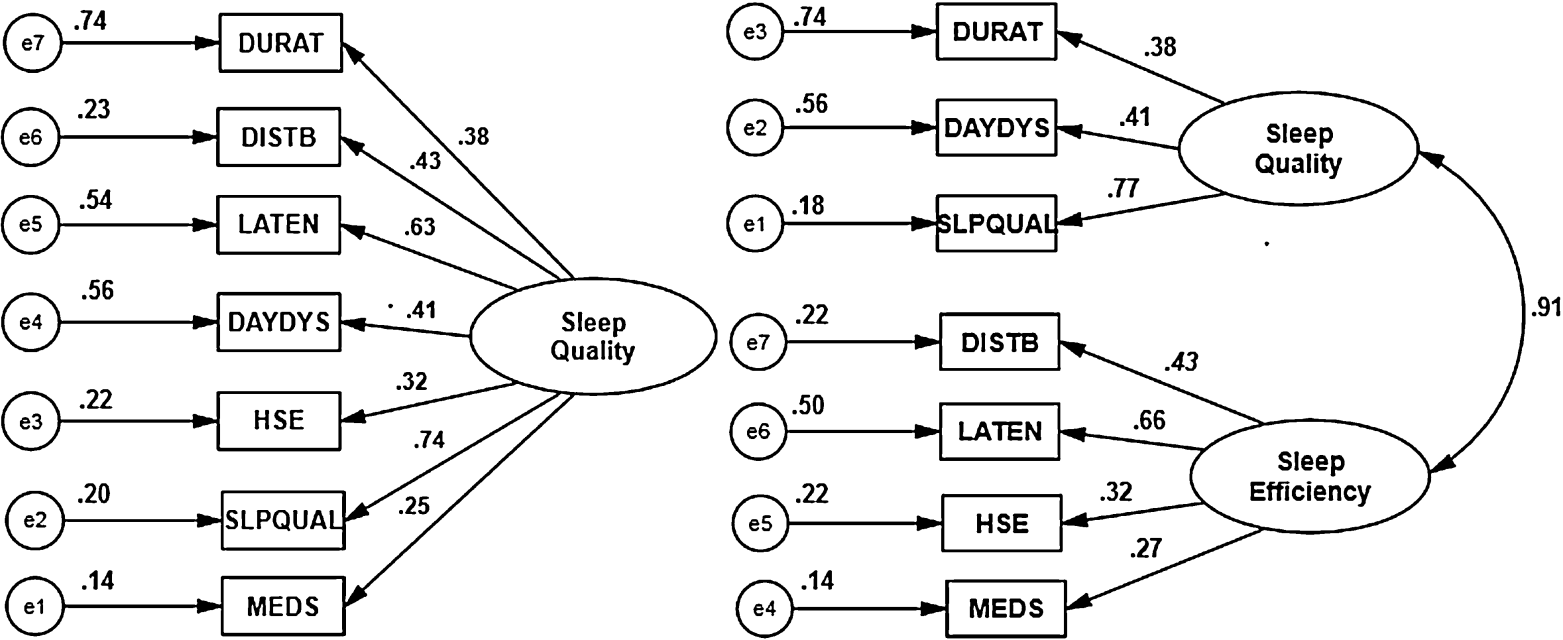
Confirmatory factor analysis models of the Pittsburgh Sleep Quality Index in the collegiate young adults. All coefficients are standardized. *Ovals* latent variables, *rectangles* measured variables, *circles* error terms, *single-headed arrows* between *ovals* and *rectangles* factor loadings, *double headed arrows* correlations, *single-headed arrows* between *circles* and *rectangles* error terms

The CFA was run on both the models (EFA outcome) (Fig. 2). The two models had an absolute fit to the data i.e. a non-significant Bollen–Stine bootstrap χ^2^ p value. The two models had similar values for all eight model fit indices i.e. GFI, AGFI, CFI, RMSEA, RMR, χ^2^, χ^2^/df and AIC (Table 4). The difference in average loadings between the models was negligible.

**Table 4:** Fit statistics of the two Pittsburgh Sleep Quality Index models in the collegiate young adults

Models	GFI	AGFI	CFI	RMSEA	RMR	χ^2^	df	p	χ^2^/df	AIC	p*
1-Factor	.982	.965	1.00	.00 (.00–.063)	.017	12.962	14	.529	.926	40.962	.691
2-Factor	.984	.965	1.00	.00 (.00–.065)	.016	12.181	13	.513	.937	42.181	.614

Goodness of fit index (GFI), Adjusted goodness of fit index (AGFI), Comparative Fit Index (CFI), root mean square error of approximation (RMSEA), root mean square residual (RMR), Akaike information criterion (AIC)

* Bollen–Stine bootstrap χ^2^ p

## Discussion

The concordant reasoning from theoretical considerations, robust measure of the factor retention, non-significant differences in the model fit indices and parsimony favor the unidimensionality of the PSQI scale in the young collegiate adults. Two previous reports have shown unidimensionality of the PSQI in other demographics. The results were established employing both EFA and CFA (Ho and Fong 2014; Rener-Sitar et al. 2014).

Certain inconsistencies between the findings of previous studies and our own do merit consideration. Our evidence for the unidimensional PSQI in the young collegiate adults is contrary to previous reports in the target population (Beavers et al. 2013; Williams et al. 2010). A study of Nigerian and Peruvian college students reported 3-factor models. While, 2-factor models were reported in students from Chile, Ethiopia and Thailand (Beavers et al. 2013; Williams et al. 2010). The 3-factor PSQI model in the Nigerian students was based only on EFA. Non-application of a more parsimonious CFA might have indicated multidimentionality (Brown 2006). No details about the factor rotation method, communality, nor criteria of factor retention were given. Moreover, 4 of the PSQI components had cross-loads above 0 > .4 (Aloba et al. 2007). None of the previous studies of collegiate students discussed communality criteria and/or advanced tests for factor retention (Beavers et al. 2013; Williams et al. 2010). The application of robust measures of factor retention, i.e. of Parallel Analysis, might have shown parsimonious models (Thompson 2004). Four model fit indices (CFI, Tucker Lewis index; TLI, RMSEA, and SRMR; Standardized root mean square residual) were employed by one of the studies, but, cut-off criteria for only three indices (CFI, RMSEA, and SRMR) were mentioned. Besides, the study presents model fit indices for the 2-factor model in the Peruvian students in spite of the EFA supporting the 3-factor model. These discrepancies complicate an independent comparison of results (Gelaye et al. 2014). The loadings of the PSQI component of sleep quality was highest in all the three models i.e. 2-factor model based on EFA (0.72), 2-factor model based on CFA (0.77) and 1-factor model based on CFA (0.74) (Table 3; Fig. 2). Moreover, removal of the PSQI component of sleep quality resulted in a maximum decrease in the internal consistency index of Cronbach’s alpha i.e. 0.65–0.55 (Table 5). Nemine contradicente, the PSQI component of medicine use contributed the lowest factor loadings in all the studies (including the present) on collegiate adults. It had a mean factor loading of 0.24 with 0.26, 0.24, 0.19, 0.28 and 0.25 in Chile, Ethiopia, Peru, Thailand and India (0.25) (our study) respectively (Gelaye et al. 2014). This redundancy in the PSQI component of medicine across ethnic divides further supports uniformity of the PSQI dimensionality among the collegiate students. The robust weighted least squares (WLS) method is more commonly used for estimation of factor loadings and/or fit indices for categorical variables but, it was not employed because it is not available in Amos. The present study does not provide a direct method for evaluating the performance of statistical models with inter-sample and intra-model differences, and/or inter-sample and inter-model differences (Manzar et al. 2016). Future studies are needed to develop direct statistical method(s).

**Table 5:** Internal consistency: Cronbach alpha and item-total statistics of the Pittsburgh Sleep Quality Index in the collegiate young adults

Pittsburgh Sleep Quality Index (PSQI) components	Alpha if item deleted
PSQI component of sleep duration	0.64
PSQI component of sleep disturbances	0.60
PSQI component of sleep latency	0.57
PSQI component of daytime dysfunction	0.63
PSQI component of sleep efficiency	0.62
PSQI component of overall sleep quality	0.55
PSQI component of sleep medication	0.64
Cronbach’s alpha of the PSQI	0.65

The unanimous outcome of the 3 tests (Scree plot-actual Eigenvalue plot, Kaiser’s criteria and cumulative variance (Fig. 1) for factor retention was a 2-factor model. But, Parallel Analysis revealed however 1-factor model of the scale (Table 3) (Thompson 2004). It has been argued that due to its robustness Parallel Analysis is a superior “best practice” test in EFA when compared to the more commonly used Kaiser’s eigenvalue-greater-than-one rule or the Scree test (Costello and Osborne 2005). Our follow up work was supportive of this view. The CFA was performed in an effort to find a parsimonious model because the Random order Eigenvalue (95th percentile) was marginally greater than the Actual Eigenvalue from Principal component analysis (PCA) for the second factor (Table 3). Similarly, CFA also helped validate the robustness of parsimony for the selected model by refuting the minor argument regarding the choice of the mean/95th percentile-as the demarcation of comparison within the distribution of randomly generated Eigenvalues (Glorfeld 1995). There was almost no difference between the Actual Eigenvalue (PCA) and the mean of the Random order Eigenvalues for the second factor (Table 3).

The correlation between the latent factors of the 2-Factor model was very strong (0.91) (Fig. 2). Therefore, it was doubtful that the two factors represented distinct constructs, i.e. they provided poor discriminant validity. The 1-factor model has the advantage of parsimony over the 2-factor model (Brown 2006). Moreover; the model fit indices did not reveal any significant difference(s) in the performance of the two models (Table 4). In conclusion, the outcome of the EFA, when taken together with the results of the Parallel Analysis, the large correlations between the two latent factors (Fig. 2), the overlapping values of model fit indices, and the parsimony of 1-factor model over 2-factor model, collectively validate the unidimensionality of the PSQI in this population of collegiate young adults.

## Abbreviations

PSQI: Pittsburgh Sleep Quality Index
BMI: Body Mass Index
EFA: exploratory factor analysis
CFA: confirmatory factor analysis
KMO: Kaiser–Meyer–Olkin
RMR: root mean square residual
CFI: comparative fit index
AIC: Akaike information criterion
GFI: goodness of fit index
AGFI: adjusted goodness of fit index
RMSEA: root mean square error of approximation
SD: standard deviation
TLI: Tucker Lewis index
SRMR: standardized root mean square residual
PCA: Principal component analysis
